# Recent progress in maintenance treatment of neuromyelitis optica spectrum disorder

**DOI:** 10.1007/s00415-020-10235-5

**Published:** 2020-10-03

**Authors:** Trygve Holmøy, Rune Alexander Høglund, Zsolt Illes, Kjell-Morten Myhr, Øivind Torkildsen

**Affiliations:** 1 Department of Neurology, Akershus University Hospital, Lørenskog, Norway; 2 Institute of Clinical Medicine, University of Oslo, Oslo, Norway; 3 Department of Neurology, Odense University Hospital, Odense, Denmark; 4 Institute of Clinical Research, University of Southern Denmark, Odense, Denmark; 5 Department of Clinical Medicine, University of Bergen, Bergen, Norway; 6 Neuro-SysMed, Department of Neurology, Haukeland University Hospital, Bergen, Norway

**Keywords:** Neuromyelitis optica spectrum disorder, Demyelinating diseases, Treatment, Monoclonal antibodies

## Abstract

**Background:**

Treatment of neuromyelitis optica spectrum disorder (NMOSD) has so far been based on retrospective case series. The results of six randomized clinical trials including five different monoclonal antibodies targeting four molecules and three distinct pathophysiological pathways have recently been published.

**Methods:**

Literature search on clinical trials and case studies in NMOSD up to July 10. 2020.

**Results:**

We review mechanism of action, efficacy and side effects, and consequences for reproductive health from traditional immunosuppressants and monoclonal antibodies including rituximab, inebilizumab, eculizumab, tocilizumab and satralizumab.

**Conclusion:**

In NMOSD patients with antibodies against aquaporin 4, monoclonal antibodies that deplete B cells (rituximab and inebilizumab) or interfere with interleukin 6 signaling (tocilizumab and satralizumab) or complement activation (eculizumab) have superior efficacy compared to placebo. Tocilizumab and rituximab were also superior to azathioprine in head-to-head studies. Rituximab, tocilizumab and to some extent eculizumab have well-known safety profiles for other inflammatory diseases, and rituximab and azathioprine may be safe during pregnancy.

## Introduction

Neuromyelitis optica (NMO) was previously characterized by bilateral optic neuritis and transverse myelitis. After the discovery of antibodies against aquaporin 4 (AQP4-IgG), it was acknowledged that clinical presentation can be more diverse, and the term NMO spectrum disorder (NMOSD) was introduced in 2007 [1]. In 2015, the International Panel for NMO Diagnosis decided to only use this unifying term [2]. In patients with AQP4-IgG, the diagnosis only requires one of the six core clinical criteria including optic neuritis and acute myelitis. In patients without AQP-4 IgG, it requires two core clinical characteristics disseminated in space, and at least one of these must be myelitis, optic neuritis, or area postrema syndrome supported by MRI [2]. Some AQP4-IgG-negative NMOSD patients may have antibodies against myelin oligodendrocyte glycoprotein (MOG-IgG). MOG-IgG-associated disease (MOGAD) is increasingly recognized as a distinct entity [3, 4], characterized by optic neuritis, transverse myelitis and/or brain stem syndrome in combination with positive MOG-IgG [5]. NMOSD with AQP4-IgG is rarely monophasic, attacks are often severe, and up to 25% have other autoimmune disease [6].

Several treatments that are effective in multiple sclerosis (MS), including interferon beta, fingolimod, alemtuzumab and natalizumab are associated with severe outcome including catastrophic exacerbations in patients with NMOSD [7–9].

**Fig. 1:**
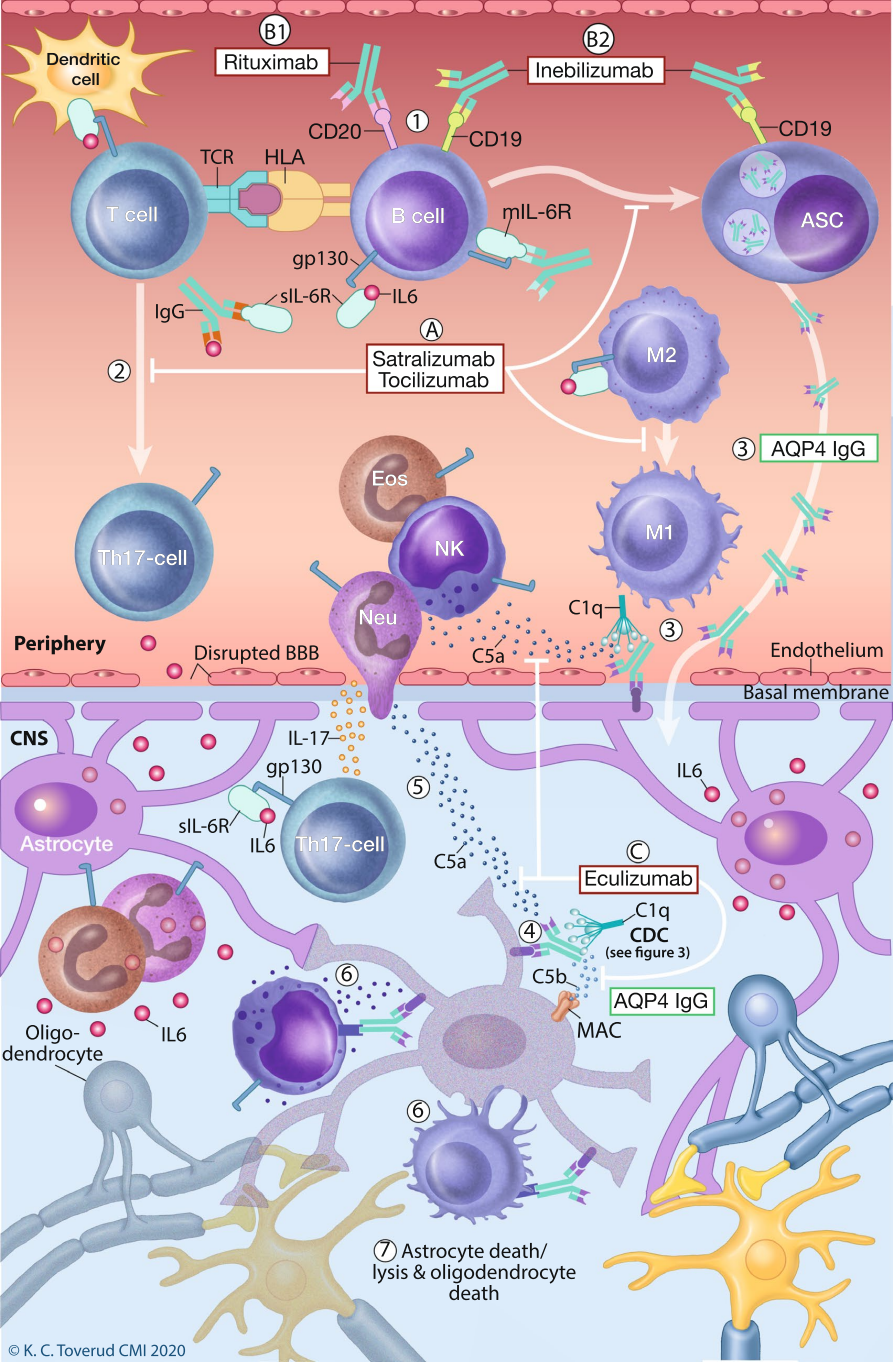
Pathogenesis and drug targets in NMOSD. Aquaporin 4 (AQP4)-specific B cells mature and differentiate in the periphery to memory cells or AQP4-IgG secreting cells (1). T cells may interact with B cells or dendritic cells, and upon stimuli including IL-6 T cells differentiate to pro-inflammatory Th17 cells that can cross the blood brain barrier (2). Inflammatory conditions allow both antibodies and complement factors to traverse the blood brain barrier and bind to AQP4 on astrocytic end feet (3). This activates complement through C1q ligation leading to formation of C5a and C5b. C5b is part of the membrane attack complexes (MAC) (4). C5a recruits pro-inflammatory leukocytes, including eosinophils, neutrophils, natural killer (NK) cells, and macrophages (5), capable of antibody dependent cellular cytotoxicity or inflammatory degranulation through Fc receptor activation (6). Astrocytes targeted by these mechanisms undergo destruction, and oligodendrocytes and neurons lose their supportive functions (7). The IL-6R blockers satralizumab and tocilizumab (A) suppress differentiation of AQP-IgG antibody secreting cells (ASC), as well as generation of pro-inflammatory Th17 T cells and M1 macrophages in favour of regulatory T cells and M2 macrophages. Rituximab (B1) kills cells expressing CD20 (mainly naïve and memory B cells, but also some T cells), while inebilizumab (B2) depletes a wider proportion of the B cell repertoire expressing CD19, including some antibody secreting cells. Both rituximab and inebilizumab deplete antigen presenting memory B cells. Eculizumab (C) blocks the complement cascade by binding complement component 5 (C5), halting generation of MAC through C5b and recruitment of pro-inflammatory cells through C5a. Printed with permission from © Kari C. Toverud

Treatment of NMOSD has been based on case series and consensus reports [10]. Very recently, the results of six randomized clinical trials with five monoclonal antibodies (Table 1) targeting four different molecules and three pathways (Fig. 1) have been published [11–16]. We here review existing and emerging NMOSD treatments about to be implemented in clinical practice. Experimental treatments were recently reviewed elsewhere [17]. AQP4-IgG is expressed on the placenta, and expecting mothers with NMOSD have high risk of miscarriage, pre-eclampsia and eclampsia, particularly if untreated or unstable [18–20]. The relapse risk during pregnancy and particularly postpartum is also high [21–24]. Compatibility with pregnancy is, therefore, important in treatment decisions and will be highlighted in this paper.

**Table 1:** Major randomized clinical trials

	RIN-1	N-MOmentum	TANGO	SAkuraStar	SAkuraSky	Prevent
Target	CD20	CD19	IL6R	IL6R	IL6R	C5
Arms	Rituximab vs placebo	Inebilizumab vs placebo	Tocilizumab vs azathioprine	Satralizumab vs placebo	Satralizumab vs placebo	Eculizumab vs placebo
Design	Double-blind	Double-blind	Open	Double-blind	Double-blind	Double-blind
Randomisation ratio	1:1	3:1	1:1	2:1	1:1	2:1
Trial duration	72 weeks	Up to 197 days	60 weeks	96 weeks	96 weeks	91 weeks
Administration	iv	iv	Iv/oral	sc	sc	iv
Baseline characteristics in the active arm						
Age (years; mean)	53	43.2	48.1	45.3	40.8	43.9
Number	19	174 analyzed	55	63	41	96
Female (%)	90	94	93	73	90	92
ARR	1.4	≥ 1	1.71	1.4	1.5	1.94
EDSS score (mean)	3.5	4.4	4.5	3.9	3.83	4.0
AQP4-IgG (%)	74^b^	87%	85	65	66	100%
Maintenance therapy^c^	Not reported	67%	98%	100%	58%	66%
Co-treatment in the active arm in study period						
Maintenance therapy^d^	None	None	Only first 12 weeks	None	Continued	Continued unless safety concerns
Prednisolone (%)	Reduced to 2 mg/day	All patients until day 21	Only rescue therapy	Only rescue therapy	41	17
Outcome (active vs comparator)						
ARR	0% vs 37%	12% vs 39% (HR 0.272) group diff: 0.31	14% vs 47% (HR 0.236) group diff: 0.29	30% vs 50% (HR 0.45) group diff: 0.6	20% vs 43% (HR 0.38) group diff: 0.46	3% vs 43%^a^ (HR 0.06) group diff: 0.07

*ARR* Annualized relapse rate, *AZA* azathioprine, *EDSS* expanded disability status scale, *MMF* mycophenolate mofetil

^a^New primary endpoint based on adjudicated relapses. ARR based on physician-determined (non-adjudicated) relapses was 0.24 (*p* < 0.001)

^b^At baseline. All patients had previously tested positive for AQP4-IgG

^c^Azathioprine or mycophenolate mofetil and rituximab

^d^Azathioprine or mycophenolate mofetil

## Traditional immunosuppressive treatment

Traditional immunosuppressive treatment, including azathioprine or mycophenolate mofetil in combination with low-dose corticosteroids has been a cornerstone in treatment of NMOSD, and is still widely used [25]. The use of low-dose corticosteroids for maintenance treatment is supported by retrospective evidence [26], but has not been studied systematically.

Azathioprine is a prodrug of 6-mercaptopurine, which inhibits purine synthesis and lymphocyte proliferation [27]. Positive results in NMOSD were first reported in 1998 in a prospective study of seven patients followed for at least 18 months [28]. Mean Expanded Disability Status Scale (EDSS) score improved from 9.0 to 3.0, and no relapses occurred.

Most evidences on azathioprine in NMOSD come from retrospective case series. Of 99 patients treated until 2009 at the Mayo Clinic, 38 discontinued treatment due to adverse events or disease activity [29]. In the remaining 61 patients, annualized relapse rate (ARR) fell from 1.5 to 0.2, and 26 became relapse-free. The failure rate for azathioprine was later reported to be higher than for rituximab and mycophenolate mofetil [30]. In 36 patients from Sao Paulo, mean ARR fell from 5.0 to 1.0 after treatment [31]. Of 100 Brazilian patients followed for median seven years, 69 had no disability progression but 11 discontinued treatment due to side effects [32]. Similar results were reported for 28 Iranian patients [33]. Of 103 AQP4-IgG-positive patients from UK, 63 remained relapse-free for a median follow-up of 18 months. Azathioprine was, however, discontinued in 47 patients, mostly because of side effects [34].

Recently, azathioprine was shown inferior to rituximab and tocilizumab on relapses and disability progression in head-to-head studies [11, 16], supporting previous case series and one open clinical trial indicating that azathioprine may be inferior to rituximab and mycophenolate mofetil [30, 35–38].

Azathioprine is transferred through placenta, but because the fetus lacks the enzyme generating active metabolites, concentrations of these are lower than in the mother [39, 40]. A recent expert recommendation emphasized that the number of exposed pregnancies exceeds 2000, and that azathioprine might be relatively safe during pregnancy [22]. Two large retrospective studies did, however, suggest increased risk of preterm birth and low birthweight [41, 42], and possibly also cardiac septal defects [42]. Another large study did not confirm this [43]. Bone marrow suppression in the infant may occur [44], and regular laboratory testing and ultrasound screening are necessary to evaluate fetal growth, organ development, and need of dose reduction [22]. The active drug is largely absent in breast milk (the maximum exposure of the drug to the infant is < 1% of the maternal dose) and blood of the infant [45, 46]. Breastfeeding is possible, although asymptomatic neutropenia has been reported [47]. The American College of Rheumatology strongly recommends continuing azathioprine during pregnancy and conditionally recommends during breastfeeding (118).

Mycophenolate mofetil is a reversible inhibitor of inosine monophosphate dehydrogenase, which is involved in guanosine nucleotide synthesis needed for lymphocyte proliferation [48]. In 2006, it was reported that a girl with NMO had been treated successfully with mycophenolate for two years [49]. The drug has later been increasingly used in NMOSD, and the first retrospective study was published in 2009 [50]. In 24 patients followed at the Mayo Clinic for a median of 28 months, median ARR fell from 1.3 to 0.09, and disability stabilized or decreased in 22 patients. Among 28 patients treated at the Mayo Clinic and the Johns Hopkins Hospital, failure rate (36%) was similar to that of rituximab and better than for azathioprine [30]. Corroborating these results, 36 of 62 Chinese patients remained relapse-free for a median of 1.5 years [51]. Case series and a meta-analysis indicate that the efficacy of mycophenolate mofetil is comparable to rituximab, and mycophenolate mofetil was also most tolerable in meta-analysis [36, 37].

Methotrexate is a folate derivative that inhibits nucleotide synthesis. In a retrospective study of 14 AQP4-IgG seropositive patients followed for a median of 21.5 months, median ARR decreased from 1.4 to 0.2, and 43% of the patients became relapse-free [52]. Disability stabilised or improved in 79%, and no patients stopped treatment due to adverse effects. In another nine patients, mean ARR dropped from 3.1 to 1.1 after treatment.[53].

Mitoxantrone intercalates into DNA causing crosslinks and strand breaks. In 2006, it was reported that five patients followed prospectively for two years had two relapses [54]. In 20 patients treated up to a cumulative dose of 120 mg/m^2^ and followed for a mean of 41 months, relapse rate decreased by 75% and disability improved or stabilized in all patients [55].

Cyclophosphamide is an alkylating agent that cross-links guanine bases in DNA. In four AQP4-IgG-positive patients, EDSS improved from 8.0 to 5.74 after treatment [56]. In another retrospective cohort of 41 patients treated for a median of 13.6 months, median ARR dropped from 0.7 to 0.0 [57]. Reports are, however, conflicting. Thus, six of seven patients treated with pulse doses of cyclophosphamide at the Federal University of São Paulo continued to have relapses from which one died [58].

Cyclosporine A is a natural immunosuppressant isolated from the fungus Beauveria nivea. In nine AQP4-IgG-positive patients NMOSD treated up to 51 months, ARR decreased from 2.7 to 0.4 [59].

Methotrexate, mitoxantrone, cyclophosphamide and mycophenolate mofetil are teratogenic [60–62], and should if possible be avoided in fertile women with NMOSD.

## Autologous hematological stem cell transplantation (HSCT)

HSCT has not been extensively used in NMOSD, and results may depend on the conditioning regime. Thus, of 16 patients with refractory disease receiving different conditioning regimens comprising either intermediate-intensity myeloablative conditioning with carmustine, etoposide, cytarabine and melphalan (BEAM) plus anti-thymocyte globulin (ATG, *n* = 9) or low-intensity non-myeloablative regimen with thiotepa-cyclophosphamide (*n* = 3) cyclophosphamide and ATG (*n* = 4), only three patients remained relapse-free and 13 had relapse or progression in disability after a median of 47 months [63]. More encouraging results have been reported for non-myeloablative cyclophosphamide, ATG and rituximab conditioning [64]. In a prospective study, 80% of 12 patients treated with this non-myeloablative regime remained relapse-free without any other immunomodulatory treatment after a median of 57 months, and most patients also became AQP-IgG seronegative [65].

## Interleukin 6 (IL-6) pathway inhibitors

The pro-inflammatory cytokine IL-6 was identified as a B cell stimulator in 1986. IL-6 is produced by several cell types including monocytes, macrophages and lymphocytes, and regulates the expression of proteins involved in inflammation, immune responses, and cell differentiation and homeostasis [66]. The IL-6 receptor (IL-6R) is expressed on the membrane of hepatocytes and leukocytes (mIL-6R), and as soluble forms (sIL-6R) in the circulation [66]. Both mIL-6R and sIL-6R transduce IL-6 signaling through glycoprotein 130 (gp130) which is ubiquitously expressed on hematopoietic and non-hematopoietic cells [67]. Classic signaling through mIL-6R induces anti-inflammatory responses, such as differentiation from M1 to M2 macrophages, while binding of sIL-6R/IL-6 to gp130 (trans-signaling) induces pro-inflammatory responses [66, 68]. In a third signaling pathway (trans-presentation), dendritic cells present mIL-6R/IL-6 to naïve T cells during differentiation into Th17 cells [69]. AQP4-specific Th17 cells are frequent in the blood of patients with NMOSD [70], and Th17 cells play an important role in the pathogenesis [71] (Fig. 1).

IL-6 levels are elevated in blood and cerebrospinal fluid of NMOSD patients and correlate with AQP4-IgG levels and disease severity [72, 73]. IL-6 facilitates disruption of the blood–brain barrier and enhances lesion severity [74, 75], and promotes AQP4-IgG production in vitro and ex vivo [76].

Tocilizumab was the first humanized monoclonal antibody against IL-6R. It is licenced for treatment of rheumatoid arthritis, giant cell arteritis and cytokine release syndrome, but has not been considered by EMA or FDA for NMOSD. Case reports suggest beneficial effects in NMOSD on relapses, disability progression, pain, and fatigue [77–80]. In 2014, a pilot study with seven NMOSD patients treated with intravenous tocilizumab reported a fall in mean ARR from 2.9 to 0.4 [81]. In another eight highly active patients followed for 10–51 months, median ARR dropped from 4.0 to 0.4 and median EDSS score from 7.3 to 5.5 [82]. Recently, similar results were reported in 12 patients treated with subcutaneous tocilizumab [83].

Tocilizumab has been used for 10 years and by more than one million rheumatoid arthritis (RA) patients, also in combination with methotrexate. The safety profile is, therefore, well established [84]. Tocilizumab induces a modest increase in lipoproteins and risk of neutropenia and bacterial infections, most markedly in combination with methotrexate [85]. This does not seem to increase with treatment duration [84, 86]. In the British Society for Rheumatology Biologics Register for Rheumatoid Arthritis, the risk of serious infections tended to be higher for tocilizumab than for rituximab [87], whereas an opposite trend appeared in Danish and Swedish RA registries [88]. So far, neutropenia and serious infections seem to be less frequent in NMOSD than in RA [77].

TANGO was an open-label, multicentre, randomised, phase-2 trial comparing intravenous tocilizumab (8 mg/kg every 4 weeks) with azathioprine (2–3 mg/kg per day) in 118 Chinese patients followed for up to 90 weeks [16] (Table 1). Almost all patients used immunosuppressive therapy at baseline. Patients randomized to tocilizumab had to stop these within 12 weeks. Patients in the azathioprine arm used azathioprine as monotherapy from week 24. Those who had used azathioprine for less than 24 weeks before randomization received supplementary immunosuppressants until 24 weeks of azathioprine treatment. Time to relapse (primary outcome) was longer in the tocilizumab than in the azathioprine group (78.9 vs 56.7 weeks; *p* = 0.0026). Eight (14%) patients on tocilizumab and 28 (47%) on azathioprine had an attack during the study, corresponding to a risk reduction of 76% (*p* < 0.0001). Among the AQP4-IgG-negative patients, two of nine (22%) on tocilizumab and three of six (50%) on azathioprine relapsed during the study. AQP4-IgG levels dropped by 50% in the tocilizumab group and remained unchanged in the azathioprine group. Tocilizumab reduced the relative risk of 24 weeks confirmed disability progression by 78% compared to azathioprine (exploratory analysis).

Overall adverse events were equally frequent, but some adverse events were more common in the azathioprine group compared to tocilizumab: elevation of alanine transferase (31% vs 46%), upper respiratory tract infections (29% vs 39%), and urinary tract infections (29% vs 36%). There were 10-grade 3–5 adverse events in the tocilizumab group and 23 in the azathioprine group. Two patients stopped tocilizumab and three patients stopped azathioprine because of adverse events. One patient on tocilizumab died from myelitis and respiratory failure, and one patient on azathioprine died from listeriosis.

The concentration of tocilizumab in cord blood serum of an infant was recently reported to be 80–90% of maternal concentrations [89]. Prospective (*n* = 180) and retrospective (*n* = 108) data on pregnancies exposed to tocilizumab (beyond the first trimester in altogether 17 patients) indicated slightly increased risks of malformation without distinct pattern, spontaneous abortion, and preterm birth [90]. One-third of the patients with adverse outcomes were, however, also treated with methotrexate/leflunomide. No evidence of increased risks was found in a Japanese cohort of 61 pregnancies including 30 patients who were treated during the first trimester [91]. NMOSD experts recently recommended that tocilizumab can be used during pregnancy in patients with very severe NMOSD, and that breastfeeding could be considered under close monitoring [22]. The American College of Rheumatology concluded that treatment until conception and breastfeeding during treatment are supported by conditional evidence [92]. Drugs and Lactation Database recommend particular caution in mothers of preterm infants [93].

**Fig. 2:**
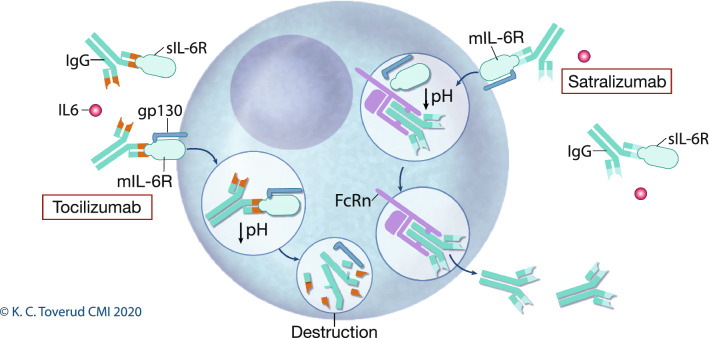
Endosomal processing of tocilizumab and satralizumab. The IL-6 receptor (IL6R) is present on a vast array of cells in the immune system, and also exists in soluble form in circulation. Membrane bound IL-6R (mIL-6R) or the soluble variant (sIL-6R) interacts with glycoprotein 130 (gp130) upon ligation with IL-6. Gp130 acts as a signal transducer into the cell that regulates expression of proteins involved in inflammation and cell homeostasis. Upon binding to mIL-6R, the receptor is brought intracellularly into endosomal compartments, where pH drops. Satralizumab, unlike the closely related tocilizumab, was specifically engineered with alterations in both variable regions to dissociate from IL-6R at low pH, and changes in the constant regions to simultaneously maintain affinity for neonatal Fc receptors (FcRn). FcRn are present in the endosomes, and allow satralizumab to recirculate to the cell surface and re-bind another s/mIL-6Rs thus enabling extended dosage protocol. Printed with permission from © Kari C. Toverud

Satralizumab is modified from tocilizumab through amino acid sequence alterations in the CDR domains, variable regions, and constant regions. The mutations in the CDR and variable regions reduce binding affinity with IL-6R at low pH (Fig. 2). Satralizumab, therefore, dissociates from IL-6R in the acidic environment found in early endosomes, and is excreted rather than being digested [94], allowing extended dosage intervals (Fig. 2). Satralizumab is approved for AQP4 NMOSD by FDA, and decision by EMA is pending.

Subcutaneous satralizumab 120 mg at week 0, 2 and every four weeks thereafter, has been tested as monotherapy and as add-on therapy in two double-blind placebo-controlled phase III trials (SAkuraStar and SAkuraSky), comprising altogether 188 patients aged 13–74 years [13, 14] (Table 1). In both trials, all reported relapses were evaluated by a clinical endpoint committee (adjudicated relapses).

In SAkuraSky, patients continued using azathioprine or mycophenolate mofetil plus oral glucocorticoids, but not rituximab [14]. At baseline, 24 of 42 patients on satralizumab and 21 of 41 patients on placebo used either azathioprine or mycophenolate mofetil. Adjudicated relapses (primary outcome) occurred in eight patients (20%) on satralizumab and in 18 (43%) on placebo (relative risk reduction 62%; *p* = 0.02). At 96 weeks, 78% of patients on satralizumab and 59% on placebo were relapse-free. The study was negative for AQP4-IgG-seronegative patients, as 5 of 14 patients on satralizumab and 6 of 14 patients on placebo relapsed. The first key secondary endpoint, reduction in pain, was negative. The number of adverse events and severe adverse events did not differ, and there were no deaths. Three patients on satralizumab and 10 patients on placebo discontinued the study during the double-blind period.

SAkuraStar was a phase-3, placebo-controlled parallel-group study of satralizumab as monotherapy [13]. Nineteen of the 63 patients (30%) receiving satralizumab and 16 of the 32 (50%) receiving placebo relapsed (HR 0.45, *p* = 0·018). After 96 weeks, 72% of patients on satralizumab and 51% on placebo were relapse-free. Again, the effect of satralizumab on ARR was only observed among AQP4-IgG-positive patients, as 10 of 22 (46%) patients on satralizumab in the AQP4-IgG-seronegative subgroup relapsed versus three of nine (33%) on placebo. As in SakuraSky, pain was not significantly reduced by satralizumab. Whereas the frequency of serious adverse events was quite similar (19% and 16%), severe adverse events were more common on satralizumab than on placebo (27% vs 6%). These were mostly considered unrelated to the study drug and did not lead to discontinuation, unless in one case of severe pneumonia in the satralizumab group. No deaths occurred in either group. One patient in each group withdrew from the study due to adverse events.

Collectively, these three trials provided evidence for the efficacy and safety of intravenous and subcutaneous anti-IL6R treatment mainly in AQP4-IgG-seropositive NMOSD, both as monotherapy and add-on treatment.

Pregnancy outcomes with satralizumab are unknown, but considerations may be similar to tocilizumab.

## Complement blocking therapy

**Fig. 3:**
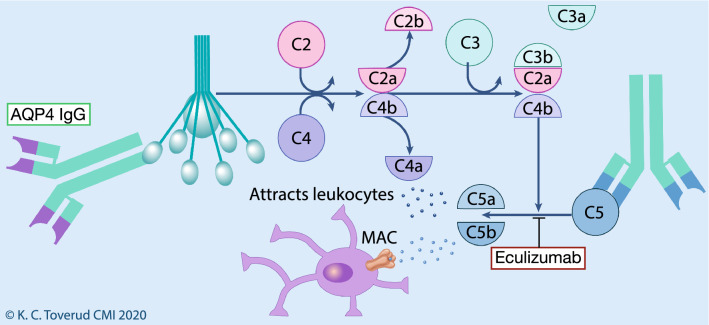
Mechanism of eculizumab. Eculizumab binds complement component 5 (C5) and prevents cleavage into C5a and C5b by C5 convertase. C5a is a potent attractant for leukocytes. C5b can form a complex with C6, and form the basis for formation of the membrane attack complex (MAC), which includes additional complement components. Printed with permission from © Kari C. Toverud

Complement deposits were early recognized in NMOSD lesions [95], and complement markedly enhances the pathogenicity of AQ4-IgG in vivo and ex vivo [74, 96–98]. Eculizumab is a monoclonal IgG2 antibody targeting C5, and inhibits cleaving and prevents release of pro-inflammatory C5a and the involvement of C5b in the membrane attack complex [99] (Fig. 3). Eculizumab could, thus, downregulate adaptive and innate immune responses either through C5a in the periphery, or through C5b on astrocytes in the CNS [100] (Figs. 1, 3). Eculizumab has been approved by EMA and FDA for AQP4-IgG positive NMOSD, and is also licenced for paroxysmal nocturnal hemoglobinuria and myasthenia gravis.

In an open-label phase II trial with 14 AQP4-IgG-positive NMOSD patients, 12 patients were relapse-free and none progressed during twelve months on eculizumab, whereas five relapsed within five months after withdrawal [101]. One patient had meningococcal sepsis and sterile meningitis during the treatment.

PREVENT was a phase III, randomized, double-blind, placebo-controlled trial comprising 143 AQP4-IgG-positive NMOSD patients randomized 2:1 to eculizumab (900 mg weekly for 4 weeks and 1200 mg every 2 weeks thereafter) or placebo [15] (Table 1). Most (76%) patients continued their previous immunosuppressive therapy. Forty-six patients had previously used rituximab, which was stopped within three months before inclusion. Given the uncertainty of when the final relapse would occur, the sponsor terminated the trial after 23 of the predefined 24 adjudicated relapses. Three of 96 patients on eculizumab and 20 of 47 on placebo had adjudicated relapse (primary outcome), corresponding to a hazard ratio 0.06 (*p* < 0.001). None of the 34 patients on eculizumab monotherapy experienced adjudicated relapses, versus 7 of 13 on placebo. Physician-determined (non-adjudicated) relapse (original primary end point) occurred in 14 patients on eculizumab and 29 on placebo (*p* < 0.001). There was no difference in disability progression as measured by EDSS (− 0.18 on eculizumab group and 0.12 on placebo). Patients receiving eculizumab had higher rates of upper respiratory tract infections and headache. There were no pneumococcus infections, but one patient on eculizumab and azathioprine died from pulmonary empyema with cultures yielding *Peptostreptococcus micros* and *Streptococcus intermedius,* which are part of the normal microbiota and common causes of opportunistic infections [102]. More patients on eculizumab (17%) than on placebo (6%) discontinued the study.

Data about eculizumab during pregnancy are available in paroxysmal nocturnal hemoglobinuria, atypical hemolytic uremic syndrome, and HELLP syndrome (hemolysis, elevated liver enzymes and low platelet levels). In these diseases, higher dose of eculizumab is required during pregnancy to block complement activity [103]. Transfer to newborns is low [103], and newborns of mothers treated with eculizumab have normal complement function [104]. The concentrations in breast milk are also low, and eculizumab is suggested to be safe during pregnancy and lactation [105]. Data are, however, limited [93], and therapeutic concentration with transient low complement levels in a newborn was recently reported [106].

## B cell depletion

B cell involvement in NMOSD may include production of autoantibodies, T cell activation and cytokine production [107] (Fig. 1).

Rituximab targets CD20 and depletes B cell lineage cells from late pro-B cells through early plamablasts, and also some T cells [108]. During the last 15 years, several retrospective case series have reported promising results in NMOSD. Rituximab has been included in treatment guidelines [109], but has not been reviewed or approved by EMA or FDA for this indication. In the first report from 2005, six of eight patients became relapse-free and seven experienced substantial disability improvement [110]. In two later retrospective studies on 25 and 23 patients, median ARR dropped from 1.7 and 1.9 to 0.0 and disability improved or stabilized in most patients [111, 112]. In the 10-year material from Johns Hopkins Hospital and the Mayo Clinic, rituximab reduced ARR up to 88.2%, and two in three patients achieved complete remission [30]. In 30 patients followed for five years in Korea, ARR fell from 2.4 to 0.3, 18 patients became relapse-free, and disability either improved or stabilized in 28 [113]. In another retrospective study of 32 patients treated first-line with rituximab, ARR was reduced by 97% [114]. Significant reduction in relapse rate was also reported in 16 children followed for a mean of 6.1 years [115].

In a 1-year open controlled trial comprising 86 patients, rituximab was significantly more effective than azathioprine [38]. ARR (primary outcome) decreased from 1.0 to 0.5 in the azathioprine group and from 1.3 to 0.2 in the rituximab group. Nineteen patients (54%) in the azathioprine group and 26 patients (79%) in the rituximab group became relapse-free. Patients receiving rituximab also improved significantly more in mean EDSS score (0.98 vs 0.44). Results were not specified for AQP4-IgG- seropositive and seronegative patients. It is, therefore, not known whether the high proportion of AQP4-IgG-seronegative patients (60.6% in the rituximab arm and 42.9% in the azathioprine arm for per protocol analysis) may have influenced the results. Any misclassification of MS as seronegative NMOSD could possibly have favoured rituximab.

In a randomized double-blind RCT (RIN-1), 19 patients were randomized to rituximab 375 mg/m^2^ weekly for four weeks and then 1000 mg every 2 week at week 24 and 48, and 19 patients were randomized to placebo [11] (Table 1). All patients were previously AQP4-IgG-seropositive, but 11 were seronegative at baseline. Mean ARR two years before inclusion was 1.4 for patients randomized to rituximab and 0.9 for patients randomized to placebo. Three patients on rituximab who discontinued treatment during the 72 weeks of follow-up (one withdrew consent, one used contraindicated drug, one had adverse event) were included in the primary analysis. Seven relapses occurred in the placebo group and none in the rituximab group (*p* = 0.0058). Change in EDSS score did not differ between groups. Eight of the 11 AQP4-IgG-seronegative patients at baseline became positive during the study. AQP4-IgG titers decreased in six patients on rituximab but in none of the patients on placebo, and increased > 10% in one patient on rituximab and in five patients on placebo.

The optimal dosage of rituximab in NMOSD is not determined. Studies using dosages ranging from 100 mg [116] to 1000–2000 mg [117] every 6 month have reported similar results. Both in adults and children, disease activity seems to correlate with depletion of B cells, but less clearly with AQP4-IgG levels [115, 118, 119]. In contrast to MOGAD, relapses mainly occur after repopulation of memory B cells in blood in AQP4-IgG NMOSD, supporting the effect of rituximab in AQP4-IgG-positive NMOSD [120]. Re-dosing based on measurements of CD27+ memory B cells may possibly allow disease control at a lower dose than fixed-dose intervals [113, 121].

Inebilizumab is a humanized, monoclonal antibody targeting the B cell surface antigen CD19. In contrast to CD20, CD19 is also expressed by pro-B cells, plasmablasts, and plasma cells, but not by any T cells. Inebilizumab has been studied in preclinical models [122] and was reported safe in phase-1 clinical studies of systemic sclerosis [123] and multiple sclerosis [124]. Inebiluzumab was approved by FDA for AQP4-IgG NMOSD June 2020, and has been granted orphan designation by EMA.

N-MOmentum was a double-blind phase-2/3 study comprising 231 patients randomized (3:1) to inebilizumab or placebo as monotherapy [12] (Table 1). The patients received intravenous inebilizumab (total dose 600 mg) or placebo on days 1 and 15, with no further doses. Two-thirds of the patients had used other immunosuppressants previously. Cyclophosphamide. Methotrexate, cyclosporine and mitoxantrone had to be stopped at least three months prior to randomization, and no other immunosupressants were allowed during the study. To minimize the risk of an attack following withdrawal of previous treatment and initiation of inebilizumab, all participants received oral prednisone 20 mg per day or equivalent from day one tapered to day 21. The double-blinded period lasted up to 197 days, or to a new NMOSD attack or termination of enrolment. All patients were thereafter offered open-label therapy.

Because of a clear demonstration of efficacy, enrolment was stopped before reaching the target of 252 patients and 67 adjudicated attacks. A total of 169 of 175 patients on inebilizumab and 54 of 56 patients on placebo completed the study period. Inebilizumab significantly increased time to onset of a new attack compared with placebo (*p* < 0.0001). In intention-to-treat analyses, 21 of 174 patients on inebilizumab experienced an attack versus 22 of 56 on placebo (hazard ratio 0.27, *p* < 0.0001). Among the 17 AQP4-IgG-seronegative patients, attacks were recorded in three of 13 on inebilizumab and none of four on placebo. Fewer patients had EDSS worsening on inebilizumab compared to placebo (*p* = 0.0049). No differences in change in low-contrast visual acuity binocular score from baseline were detected, but significantly fewer inebilizumab-treated patients experienced optic neuritis compared to placebo (10 patients in each group, corresponding to HR 0.288). Patients on inebilizumab  had fewer new MRI lesions (*p* = 0.0034) and hospitalizations (*p* = 0.010) compared to placebo.

Adverse events, serious adverse events, infusion reactions and infections were equally frequent among patients on inebilizumab and on placebo, and no malignancies were reported. No death occurred during the placebo-controlled phase, but two patients died during the open-label phase, one (originally receiving placebo) because of respiratory insufficiency due to the ongoing disease process. Another patient (originally receiving inebilizumab) developed new neurological symptoms including seizures nine days after receiving 300 mg inebilizumab in the open-label period. MRI showed new large lesions in white and grey matter, considered not representative for progressive multifocal leukoencephalopathy (PML). PCR for John Cunningham virus in cerebrospinal fluid were negative in two laboratories but positive in another. No definitive diagnosis was made, and it could not be excluded that the death was treatment-related.

Monoclonal antibodies are not transported to the fetus until the second trimester [125]. Given their prolonged effect on B cells and half-life around three weeks [126], rituximab and inebiluzumab administered before conception may protect the mother against relapses without exposing the fetus to potential harmful effects. Data from 153 pregnancies in mothers treated with rituximab for hematological malignancies and autoimmune diseases, including 21 treated during pregnancy, indicated increased risk of miscarriage and premature birth [127]. Many of these mothers had, however, also used other immunosuppressants. Limited data in MS and NMOSD do not suggest increased risk of adverse pregnancy outcomes beyond reversible hematological abnormalities [128, 129], also in those very few treated during pregnancy [129, 130]. Both the American College of Rheumatology and NMOSD experts conditionally recommend rituximab until conception, and if necessary also during pregnancy [22, 92]. B cells should be measured in the infant, and vaccination planned accordingly.

Rituximab concentrations in breast milk are low, and rituximab is likely not absorbed from the gastrointestinal tract [131, 132]. The American College of Rheumatology recently concluded that recommending breastfeeding during treatment with rituximab is supported by strong evidence [92]. Drugs and Lactation Database recommend caution, particularly in preterm infants [133].

Pregnancy outcomes with inebilizumab are unknown, but considerations may be similar to anti-CD20 therapy.

## Discussion

Differences in study design (active comparator versus placebo, monotherapy versus add-on therapy, open versus blinded) preclude conclusions on the relative efficacy of the monoclonal antibodies in NMOSD. Patients in the RIN-1 study of rituximab, N-MOmentum  of inebiluzumab and SakuraSTAR of satralizumab had to stop ongoing immunosuppressive treatment [11–13]. The use of prednisolone was also restricted in these studies and in the TANGO study of tocilizumab (Table 1). It is, therefore, conceivable that control patients in these studies had poorer outcome than they would have had in routine practice [134]. On the other hand, co-treatment with immunosupressants that has not been proven effective in randomized trials may possibly have confounded the results of the PREVENT study of eculizumab and the SAkuraSky study of satralizumab [100]. It is, therefore, reassuring that satralizumab was superior to placebo also as monotherapy [13], and that rituximab and tocilizumab were more effective than azathioprine [16, 38]. Data on de novo treatment are scarce in NMOSD, particularly for the novel drugs, as all or more than two-thirds of the patients in the recent clinical trials had used other immunosuppressants at enrolment.

The robust treatment responses to eculizumab and to satralizumab monotherapy compared to placebo prove that IL-6 signaling and complement activation are relevant therapeutic targets in NMOSD patients with AQP4-IgG, who have the highest risk of relapse [135]. The effect of these novel treatment options is, however, less clear for patients without AQP4-IgG. Eculizumab was only tested in AQP4-IgG-positive patients [15], and the effect of satralizumab was not convincingly shown in seronegative patients [13, 14].

In MS patients, inebilizumab induced a 10.5% decrease in total immunoglobulin levels after 24 weeks and 15.0% after 18 months [126], exceeding that recorded for rituximab [136].

It could be speculated that the broader depletion of the B cell linage by inebilizumab, including antibody secreting cells, could be an advantage compared to rituximab [134]. Although theoretically appealing in an autoantibody-mediated disease, this remains to be proven. More pronounced drop in immunoglobulins may also lead to more infections.

Anti-drug antibodies may reduce therapeutic effects and can also cause adverse reactions through formation of immune complexes. This is particularly relevant for B cell therapies, as rituximab is a chimeric, whereas inebilizumab is a humanized antibody. Whereas no data exist in NMOSD, about one-third of MS patients develop antibodies against rituximab [137]. The clinical effect of such antibodies is not firmly established. They are, however, associated with poorer B cell depletion [137], which likely is unfavorable in NMOSD [118, 119], and may rarely also cause serum sickness [138]. The immunogenicity of inebilizumab seems much lower. Anti-inebilizumab antibodies were measurable in only 3% of NMOSD patients on inebilizumab, which was not more frequent than in patients on placebo [12]. Anti-drug antibodies are rare in rheumatoid arthritis patients treated with tocilizumab [139]. It is not known whether modifications have increased the immunogenicity of satralizumab.

Complement blocking increases the risk of meningococcal and encapsulated bacterial infection [140–142]. Therefore, patients in PREVENT and also the REGAIN study of eculizumab in myasthenia gravis were vaccinated against *Neisseria meningitidis*, and meningitis was not reported in either study [143]. Long-term treatment with rituximab and possibly also tocilizumab and satralizumab are also associated with infection risk [144, 145], and pneumococcal vaccination is often recommended before starting rituximab. Vaccination may, however, increase relapse risk in untreated NMOSD patients [146]. On the other hand, eculizumab, rituximab and most likely also inebilizumab reduce vaccine responses [142, 147]. Whether or not to start treatment before vaccination must be based on individual evaluation of infection and relapse risk.

## Conclusion

The therapeutic armamentarium for NMOSD has expanded, and now includes evidence-based oral, subcutaneous and intravenous medications given daily, biweekly, monthly and every 6 months. The efficacy of monoclonal antibodies in NMOSD-IgG-positive patients is better documented, including class I evidence for eculizumab, inebilizumab and satralizumab, and likely also greater than traditional immunosuppressive therapy with azathioprine. Based on current evidence, we suggest that most NMOSD patients, particularly those with AQP4-IgG, should start with one of the monoclonal antibodies as a first-line treatment. Currently, we do not know which of these offers the best efficacy. Thus, treatment decisions will depend on factors like availability, price, co-morbidity and future pregnancy planning. For women who might become pregnant, rituximab has the best documented and possibly also most favorable safety profile, but azathioprine and some other monoclonal antibodies may also be compatible with pregnancy in selected patients.

Given the rarity of NMOSD, head-to-head studies of the monoclonal antibodies will not likely be conducted. Registry-based follow-up and real-life life studies will hopefully clarify optimal sequencing and combination, and particularly for B cell depleting drugs also long-term dosing regimens.
